# Real-world economic impact of onabotulinumtoxina in patients with chronic migraine

**DOI:** 10.1186/1129-2377-1-S14-P216

**Published:** 2013-02-21

**Authors:** JF Rothrock, LM Bloudek, TT Houle, D Andress-Rothrock, C Hanlon, SF Varon

**Affiliations:** 1 The University of Alabama School of Medicine, Birmingham, AL, USA; 2 Allergan, Inc., Irvine, CA, USA; 3 Wake Forest University School of Medicine, Winston-Salem, NC, USA; 4 University of Alabama Headache, Treatment and Research Program, Birmingham, AL, USA; 5 Allergan, Inc., Irvine, CA, USA, UK

## Introduction

Compared to episodic migraine, chronic migraine (CM) is associated with greater disability, worse quality of life, and higher costs related to healthcare resource use (HRU). OnabotulinumtoxinA can be used effectively for headache prophylaxis in CM patients with CM, but the effect of treatment on HRU is unknown.

## Objective

Quantify the reduction in migraine-related HRU among CM patients treated for 6 months with onabotulinumtoxinA.

## Methods

We analyzed data from 223 CM patients who presented to a university-based headache specialty clinic in Birmingham, AL between January 2007 and April 2011 and who were treated with 2 cycles of onabotulinumtoxinA (155-195U per cycle). Frequencies of migraine-related hospitalization and utilization of emergency departments (EDs) or urgent care centers for acute migraine treatment over the 6 months preceding initial treatment with onabotulinumtoxinA were collected retrospectively. Migraine-related HRU data following initial treatment were recorded prospectively. Change in HRU after onabotulinumtoxinA was assessed using paired student’s t-test with á=0.05. Costs of treatment and HRU were based on the 2011 Medicare physician fee schedule and publicly available national ED and hospital costs (HCUP.net). The cost of an urgent care visit was approximated to be 1/4 the cost of an ED visit. The estimated cost for two cycles of onabotulinumtoxinA therapy (including physician administration fee and two 100U vials, to account for wastage) was $2601.

## Results

Patients demonstrated a mean reduction of 0.92 ED visits (p<0.001), 0.33 urgent care visits (p<0.001), and 0.11 hospitalizations (p=0.003) following initiation of treatment; application of conservative national estimates for related costs yielded a reduction of $1025 per patient. The reduction in HRU offset 39% of the estimated cost for 6 months of onabotulinumtoxinA.

## Conclusions

A reasonable proportion of the cost of onabotulinumtoxinA treatment for CM may be offset by a reduction in migraine-related ED visits, hospitalizations, and urgent care visits. Support: Allergan, Inc.

